# *Chlamydia trachomatis* Infection Is Associated with E-Cadherin Promoter Methylation, Downregulation of E-Cadherin Expression, and Increased Expression of Fibronectin and α-SMA—Implications for Epithelial-Mesenchymal Transition

**DOI:** 10.3389/fcimb.2017.00253

**Published:** 2017-06-14

**Authors:** Jovana Rajić, Aleksandra Inic-Kanada, Elisabeth Stein, Svetlana Dinić, Nadine Schuerer, Aleksandra Uskoković, Ehsan Ghasemian, Mirjana Mihailović, Melita Vidaković, Nevena Grdović, Talin Barisani-Asenbauer

**Affiliations:** ^1^Department of Molecular Biology, Institute for Biological Research “Siniša Stanković”, University of BelgradeBelgrade, Serbia; ^2^OCUVAC – Center of Ocular Inflammation and Infection, Laura Bassi Centres of Expertise; Center for Pathophysiology, Infectiology and Immunology; Medical University of ViennaVienna, Austria

**Keywords:** *Chlamydia trachomatis*, human conjunctival epithelial cells, HCjE, epithelial-mesenchymal transition (EMT), DNA methylation, E-cadherin

## Abstract

*Chlamydia trachomatis* (*Ct*) can induce scarring disease of the ocular mucosa, known as trachoma, the most common infectious cause of blindness worldwide. We hypothesized that epithelial-mesenchymal transition (EMT) contributes to the fibrotic process in trachomatous scarring. Infection of human conjunctival epithelial cells (HCjE) with *Ct* activated signaling pathways involved in EMT induction, which was correlated with decreased expression of E-cadherin, guardian of the epithelial phenotype. In addition, *Ct* infection was associated with increased expression of two mesenchymal cell markers: fibronectin and α-SMA. The DNA methylation statuses of selected regions of E-cadherin, fibronectin, and α-SMA genes revealed that *Ct* infection was accompanied with changes in DNA methylation of the E-cadherin promoter, while the expression of the two mesenchymal markers was not related with this epigenetic event. Our data suggest that *Ct* infection of conjunctival epithelial cells induces EMT-like changes that go along with modification of the methylation profile of the E-cadherin promoter and could, as one of the earliest events, contribute to processes triggering conjunctival scarring.

## Introduction

Trachoma is the most common cause of preventable blindness from infectious origin worldwide (Taylor et al., 2014). An estimated 200 million people live in endemic areas and are at risk of developing trachoma-related blindness (WHO, 2014, 2016). The disorder is triggered by ocular infection with ocular strains of *Chlamydia trachomatis* (*Ct*) via direct smear infection or mechanical transmission (Solomon et al., 2004; Wright et al., 2008). Conjunctival infection (often repeated) precedes chronic inflammation and eventually leads to conjunctival fibrosis (Whittum-Hudson et al., 1986; Hu et al., 2013). The resulting scarring is the key element in the development of the blinding sequelae leading to inversion of the eyelids and lashes (trichiasis) that wound the ocular surface (Mohammadpour et al., 2016). Chronic keratoconjunctivitis and/or prolonged trichiasis cause corneal opacification and blindness (Mabey et al., 2003; Brunham and Rey-Ladino, 2005; Wolle et al., 2009). Like in any fibrotic process, it is believed that the scar tissue in trachoma originates from activated fibroblasts (Kechagia et al., 2016), and there is supporting evidence that innate and adaptive immunity involved in the resolution of *Ct* infection may initiate the fibrotic process (Brunham and Rey-Ladino, 2005). Thus, to date, research has been focused on elucidating the fibroblast-stimulating profibrotic mediators. Only recently, an alternative pathway, the process of epithelial-mesenchymal transition (EMT), was proposed to be involved in the development of end-stage trachoma (Derrick et al., 2015).

EMT consists of a series of events constituting reversible transition of epithelial into mesenchymal cells. During EMT, cells lose their epithelial characteristics, such as apical-basal polarity and cell–cell junctions, and acquire mesenchymal features, including front-back polarity, enriched cell–matrix interactions, and motility. Changes in cell morphology and function during EMT can be traced through numerous markers established in *in vitro* models of EMT where complete transition from the epithelial to the mesenchymal state is observed (Zeisberg and Neilson, 2009). However, data from *in vivo* studies indicated that EMT comprises a whole spectrum of intermediary, transitional states between the epithelial and the mesenchymal phenotype (Nieto et al., 2016). In fact, the presence of intermediary epithelial and mesenchymal phenotypes had been observed in most of the EMT-related processes and marked as “EMT-like,” “incomplete,” or “partial” EMT (Jordan et al., 2011; Morbini et al., 2011; Grigore et al., 2016). EMT has several triggers, which all act via different pathways. TGFβ family members are considered the main inducers of EMT. TGFβ signaling results in the activation of either SMAD transcription factors in the canonical pathway, or activation of PI3K-AKT and MAP kinases in the non-canonical pathway (Miyazono, 2009). These kinases also activate downstream tyrosine kinase receptors (RTKs), through which the other growth factors act (Lamouille et al., 2014). Another major signaling pathway involved in EMT is the Wnt pathway, which involves inhibition of GSK3β and consequent induction of β-catenin-regulated gene expression (Niehrs, 2012). Besides soluble ligands, it has been shown that components of the extracellular matrix (ECM) are important for EMT induction through integrin receptors (Chen et al., 2013). The activation of different EMT-inducing pathways and their intensive crosstalk result in the induction and activation of the same set of EMT-related transcription factors (ZEB, SNAIL, and TWIST) and the expression of common EMT marker genes. Thus, decreased expression of E-cadherin, a cell–cell adhesion molecule that is highly expressed in epithelial cells, has become a hallmark of the EMT process (Zeisberg and Neilson, 2009). A frequently used mesenchymal EMT marker is increased α-SMA expression, as it appears *de novo* in response to tissue injury, partially as a result of the EMT process. In addition, fibronectin, a glycoprotein responsible for cellular interactions with the ECM, is of particular interest, as its expression increases during EMT, and it has been shown to be one of the EMT inducers through integrin signaling (Kim et al., 2006).

EMT is regulated at the transcriptional, posttranscriptional (through miRNA and alternative splicing), and posttranslational (through numerous stability- and activity-affecting protein modifications) levels. Recent findings have suggested that epigenetic events are master regulators of expression of all EMT-related genes (Tam and Weinberg, 2013; Serrano-Gomez et al., 2016). The effect of DNA methylation on gene expression has been shown for several EMT marker genes (Lombaerts et al., 2006; Hu et al., 2010), while the overall significance of DNA methylation for EMT was confirmed by DNA methylomes of cells undergoing EMT, which revealed that changes in DNA methylation of both promoters and gene bodies are dynamic and reversible and are strongly associated with transcriptional regulation of EMT-related genes (Carmona et al., 2014).

Possible factors giving rise to conjunctival fibrosis are infection/inflammation, trauma, potential co-infections, dust, and genetics. Various bacteria and viruses are known to induce epigenetic changes in host cells (Minarovits, 2009; Paschos and Allday, 2010; Niller and Minarovits, 2016), prompting researchers to speculate that *Ct* might directly influence epigenetic mechanisms involved in EMT processes in trachoma (Derrick et al., 2015). In genital models, Igietseme et al. (2015) were able to show that *Ct* infection *per se* induced EMT *in vitro* and *in vivo*, which was associated with altered expression of crucial miRNAs that control EMT and fibrosis. Nevertheless, the underlying mechanisms by which *Ct* might induce the development of fibrosis have not been fully elucidated.

The aim of this study was to test the ability of *Ct* to induce EMT-related processes in human conjunctival epithelial (HCjE) cells *in vitro* by analyzing the expression of selected relevant EMT inducers and marker genes (E-cadherin, fibronectin, α-SMA), and to investigate whether alterations in gene expression after *Ct* infection correlate with changes in their DNA methylation profiles and subsequent gene expression. The involvement of epigenetic mechanisms in EMT process in trachoma would open up possibilities for novel therapeutic approaches in trachoma treatment.

## Materials and methods

### Infection of HCjE cells with *Ct*

All experiments involving biohazards (*Ct* and cell lines) or hazardous chemicals were carried out using standard protective measures and following all local and national regulations.

Stocks of *Ct* (serovar B/HAR-36, ATCC® VR-573™) were grown in McCoy cells (ATCC® CRL-1696™) according to standard procedures (Rank et al., 1988). Briefly, confluent cultures of McCoy cells were inoculated at an multiplicity of infection (MOI) of 10 in 75-cm^2^ tissue culture flasks, centrifuged at 900 × *g* for 1 h, and incubated for 48 h in the presence of 1 μg/ml cycloheximide (Sigma, Steinheim, Germany). The cells were disrupted with glass beads and centrifuged at 900 × *g* for 15 min to remove host cell debris. Inoculation stocks and McCoy cells were tested to be negative for mycoplasma.

hTert-immortalized HCjE cells, kindly provided by Prof. Ilene Gipson (Schepens Eye Research Institute, Harvard Medical School, Boston), were maintained in keratinocyte serum-free medium (Life Technologies, Paisley, UK) supplemented with bovine pituitary extract, 0.2 ng/mL recombinant human epidermal growth factor, 0.4 mM CaCl_2_, and 1000 U/mL penicillin and 1000 μg/mL streptomycin (GE Healthcare, South Logan, UT, USA) at 37°C, under 5% CO_2_ and 95% humidity. The medium was changed every second day, and the cells were passaged at 70% confluence. Cells were harvested by trypsinization (0.05% trypsin/0.02% EDTA in PBS, GE Healthcare) and seeded at a density of 400,000 cells/flask in 75-cm^2^ flasks. Confluent cultures (an estimated 3.5 × 10^6^ HCjE cells) were infected with 10^7^ infectious units (IFU) *Ct* in inoculation medium (DMEM/Ham's F-12 1:1, Life Technologies) per flask and the MOI was calculated to be 3. To promote adhesion, the flasks were centrifuged at 900 × *g* for 1 h at 37°C. After incubation for 1 h at 37°C, the inoculation medium was changed to keratinocyte serum-free medium without antibiotics, and the cells were incubated for another 72 h. Host cell viability was monitored by lactate dehydrogenase assay and immunoblot analysis for PARP-1, procaspase 3, and active form of caspase 3; no differences were observed between control and *Ct*-infected cells.

### Isolation of DNA, RNA, and proteins

Cells were harvested by scraping and pelleted at 900 × *g*. For isolation of DNA, RNA, and proteins, an AllPrep DNA/RNA/Protein Mini Kit (Qiagen, Hilden, Germany) was used according to the manufacturer's instructions. Samples were stored at −80°C until analysis.

### Quantitative reverse-transcription PCR (qRT-PCR)

Reverse transcription was carried out with the RevertAid First Strand cDNA Synthesis Kit (Fermentas, Burlington, Canada) on 1 μg DNAse I-treated RNA, using oligo(dT) primers. qRT-PCR was carried out using Maxima SYBR Green/ROX qPCR Master Mix (Fermentas) on a QuantStudio 3 Real-Time PCR system (Applied Biosystems, Carlsbad, CA, USA). The thermal cycles used were as follows: initial denaturation at 95°C/10 min and 40 cycles of two-step PCR at 95°C/15 s and 57°C/60 s (all genes except *SNAI1* and *ZEB2*) or 60°C/60 s (*SNAI1* and *ZEB2*). The relative expression levels of target genes were calculated using the comparative 2^−ΔΔCt^ method after normalization using *GAPDH* as an endogenous control, as no difference in *GAPDH* mRNA expression between control and *Ct*-infected HCjE cells was observed.

Primers were designed with Primer-BLAST (https://www.ncbi.nlm.nih.gov/tools/primer-blast/) using sequences stored in GenBank under the following accession numbers: *TGF*β*1* (transforming growth factor beta 1) NM_000660; *TGF*β*2* (transforming growth factor beta 2) NM_003238; *CDH1* (cadherin 1) NM_004360; *FN1* (fibronectin 1) NM_212482; *ACTA2* (actin, alpha 2, smooth muscle, aorta; α-SMA) NM_001141945; *SNAI1* (snail family transcriptional repressor 1) NM_005985; *ZEB2* (zinc finger E-box binding homeobox 2) NM_014795; *GAPDH* (glyceraldehyde-3-phosphate dehydrogenase) NM_002046. The primer sequences are listed in Table S1 (Supplemental Information).

### Immunoblot analysis

Equal amounts of cell lysates were separated by 12% sodium dodecyl sulfate polyacrylamide gel electrophoresis and transferred onto polyvinylidene difluoride membranes. Immunoblot analysis was conducted using antibodies listed in Table S2 (Supplemental Information). All primary antibodies were incubated overnight at 4°C, followed by incubation with the appropriate horseradish peroxidase-conjugated secondary antibody at room temperature for 1 h. Blots were stained using chemiluminescence according to the manufacturer's instructions (Amersham Pharmacia Biotech, Amersham, UK). Membranes were stripped and reprobed with anti-GAPDH antibody as a loading control. Signal intensities were analyzed using TotalLab ver. 1.10 electrophoresis software (Phoretix International Ltd, Newcastle upon Tyne, UK) and normalized to GAPDH as an internal control, as no difference in GAPDH protein expression between control and *Ct*-infected cells was observed.

### Immunocytochemistry

Cells were grown on sterile glass coverslips in 24-well tissue culture plates and infected with 10^5^ IFU *Ct* per well. Infection was carried out as described above. After 72 h, the cells were fixed with 4% paraformaldehyde (Science Services GmbH, Munich, Germany) in PBS for 15 min at 37°C. The cells were permeabilized with PBS containing 0.3% Triton X-100 for 10 min and blocked with 3% bovine serum albumin for 1 h. The coverslips were incubated overnight at 4°C with primary antibodies listed in Table S2 and diluted in PBS containing 0.2% Tween-20. For acquiring fluorescent signal, the slides were incubated overnight at 4°C with fluorescently labeled secondary antibodies listed in Table S2. All washes were done in PBS-Tween-20 0.2% (v/v). DNA was visualized by adding 4,6-diamidino-2-phenylindole (DAPI) (Roche Diagnostics, Mannheim, Germany) (0.2 μg/mL) for 20 min. The cover slips were mounted on glass slides with Mowiol (Calbiochem, San Diego, CA, USA) and images were taken with an Axiocam digital camera attached to the Axio Observer Z1 microscope (Carl Zeiss Microscopy GmbH, Jena, Germany), using appropriate filters.

### DNA methylation analysis

#### Bisulfite conversion of DNA

Genomic DNA isolated from control and *Ct*-infected cells was bisulfite-converted using the EZ-DNA methylation kit (D5002; Zymo Research, Orange, CA, USA) according to manufacturer's instructions. CpG islands (short stretches of DNA in which the frequency of CG dinucleotides is higher than in other regions) were predicted using CpG Island Searcher (http://cpgislands.usc.edu/) (Takai and Jones, 2003) with standard parameters (%GC, 55; Obs CpG/Exp CpG, 0.65; Length, 500 bp; Distance, 100 bp). Genomic DNA sequences that were uploaded for analysis encompassed the regions 3,000 bp upstream and 3,000 bp downstream from the transcription start site (TSS) (marked as +1) of the E-cadherin (*CDH1*; NCBI ref. sec. NG_008021.1), fibronectin (FN1; NG_012196.1), and α-SMA (*ACTA2*; NG_011541.1) genes. All primer pairs, methylated and unmethylated, for DNA methylation analysis were designed in MethPrimer (http://www.urogene.org/cgi-bin/methprimer/methprimer.cgi) and are listed in Table S3 (Supplemental Information).

#### Methylation-specific PCR (MSP)

MSP analysis was conducted using methylated (M) primers containing two CpGs in each primer (forward and reverse) for *CDH1*, and one CpG in forward and two CpGs in reverse primers for *FN1* and *ACTA2*. The reaction mixture for MSP contained Maxima SYBR Green/ROX qPCR Master Mix (Fermentas), 4 μL of bisulfite-converted DNA (theoretical concentration of 60 ng/μL), and 0.5 μM of M primers in a final volume of 10 μL. MSP was conducted on the QuantStudio 3 Real-Time PCR system. PCR cycling conditions were as follows: initial denaturation step at 95°C/10 min followed by 40 cycles of denaturation at 95°C/15 s and annealing and elongation at 58°C/60 s for *CDH1* and *FN1* or 55°C/60 s for *ACTA2*. Reference primers, which do not contain CpGs and exclusively amplify bisulfite-converted DNA, were used as an endogenous control for normalization of the MSP results and as a control for efficient bisulfite conversion. After normalization, the amount of the methylated product was estimated in comparison to *in vitro* methylated DNA, bisulfite-converted and amplified under the same conditions, which was assumed to be 100% methylated. *In vitro* methylation was performed using CpG Methylase M.SssI (E2011) (Zymo Research, Orange, CA, USA), which completely methylates all cytosine residues.

#### Methylation-sensitive high-resolution melting (MS-HRM)

Human methylated and non-methylated DNA standards (D5014) (Zymo Research) were bisulfite-converted according to manufacturer's instructions. To generate a range of methylated:non-methylated DNA standards, methylated and non-methylated templates were mixed at 0:100, 25:75, 50:50, and 100:0 ratios. PCR amplification and MS-HRM analysis were carried out sequentially on a QuantStudio 3 Real-Time PCR system. Multiplex PCR with a mix of all four primers (M and unmethylated, U) was used to cover all possible variants in methylation status. PCR was carried out in a 10-μL reaction mixture consisting of 5 μL 2 × MeltDoctor HRM Master Mix (Applied Biosystems), 0.15 μM of each primer, and 1 μL bisulfite-converted template (theoretical concentration of 20 ng/μL). The amplification consisted of initial denaturation at 95°C/10 min, followed by 45 cycles of three-step PCR and a final elongation step at 72°C/7 min. The three-step PCR program consisted of 5 cycles of denaturation at 95°C/30 s, annealing at 58°C/30 s and elongation at 72°C/60 s; 5 cycles of denaturation at 95°C/30 s, annealing at 53°C/30 s and elongation at 72°C/60 s; and 35 cycles of denaturation at 95°C/30 s, annealing at 47°C/30 s and elongation at 72°C/60 s. The HRM program consisted of temperature ramping from 60–95°C by 0.025°C/s with florescence acquisition at each temperature increment. HRM Software v3.1 (Applied Biosystems) was employed for end-product analysis. Peak heights of standards, obtained from difference curves aligned against an unmethylated control (0%), were plotted against the percentage of methylation to obtain a standard curve and linear regression equation. After normalization, the degree of methylation of each sample was quantitatively calculated from the linear regression equation.

#### Bisulfite sequencing

Bisulfite-modified DNA was amplified using M primers in a 30-μL reaction mixture that included 15 μL 2 × AmpliTaq Gold 360 Master Mix (Applied Biosystems), 0.5 μM of each primer, and 4 μL of bisulfite-converted template (theoretical concentration of 80 ng/μL). Amplification was carried out on an Eppendorf Mastercycler Pro (Eppendorf Austria GmbH, Vienna, Austria), using the following thermal cycles: initial denaturation at 95°C/10 min, 45 cycles of three-step PCR, and final elongation at 72°C/7 min. The three-step PCR program consisted of 5 cycles of denaturation at 95°C/30 s, annealing at 62°C/30 s and elongation at 72°C/60 s; 5 cycles of denaturation at 95°C/30 s, annealing at 57°C/30 s and elongation at 72°C/60 s; and 35 cycles of denaturation at 95°C/30 s, annealing at 51.5°C/30 s and elongation at 72°C/60 s. Purification of PCR products and Sanger sequencing of bisulfite-converted DNA were carried out by Eurofins Genomics AT (Vienna, Austria). The sequencing results were analyzed using the BiQ Analyzer software (Max Planck Institute for Informatics, Saarbrücken, Germany).

### Statistical analysis

Experiments were performed in triplicate and all results are presented as the mean ± SD. IBM SPSS Statistics for Windows, Version 20.0 (Armonk, NY, USA) was used for statistical analysis. The Shapiro–Wilk test was used to determine whether samples followed normal distribution. For normally distributed data, two groups were compared using Student's *t*-test. For data with non-normal distribution, the Mann–Whitney U test was applied. The null hypothesis was rejected at *p* < 0.05. “^*^” Indicates statistically significant difference compared to control cells (^*^p < 0.05; ^**^p < 0.01).

## Results

### *Ct* infection triggers EMT-inducing signaling pathways

**Figure 1:**
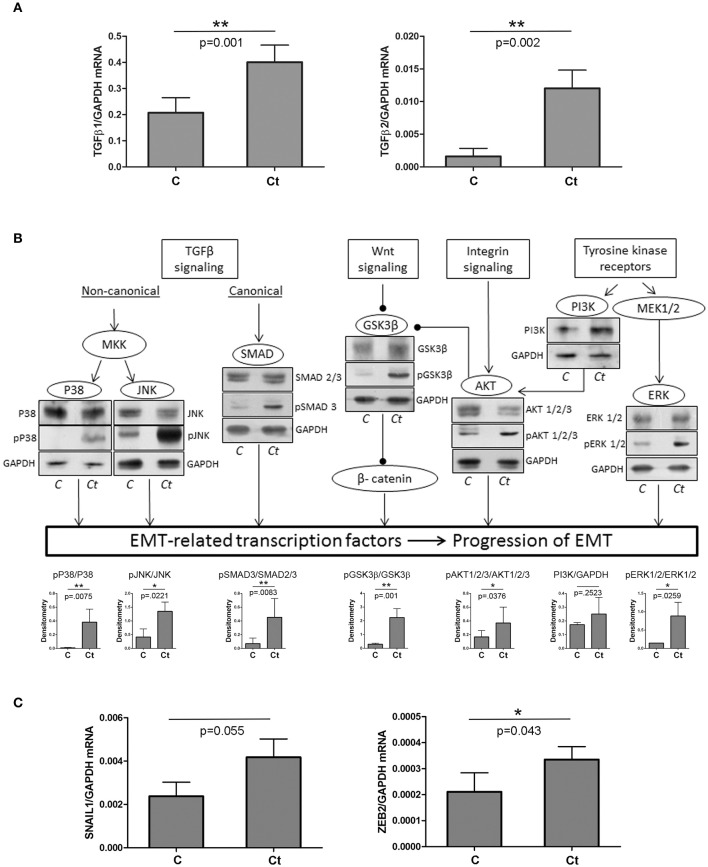
*Ct* Infection Triggers EMT-inducing Signaling Pathways. **(A)** mRNA levels of *TGF*β*1* and *TGF*β*2* in control (C) and HCjE cells infected with 10^7^ IFU of *Ct* (Ct) as determined by qRT-PCR. mRNA levels are relative to GAPDH and are expressed as means ± SDs. **(B)** Overview of signaling pathways involved in EMT induction and the presence of key components as determined by immunoblot analysis in control (C) and HCjE cells infected with 10^7^ IFU of *Ct*. Quantitative results of at least three immunoblots are presented. The graph depicts changes in phosphoprotein levels relative to the corresponding non-phosphorylated proteins, after normalization to GAPDH, which was used as loading control. The results are expressed as means ± SDs. **(C)** mRNA levels of *SNAIL1* and *ZEB2* in control (C) and HCjE cells infected with 10^7^ IFU of *Ct* as determined by qRT-PCR. mRNA levels are relative to that of *GAPDH* and are expressed as means ± SDs.

First, we tested the hypothesis that infection of HCjE with *Ct* induces changes in expression profiles of proteins that could trigger a transition of the conjunctival epithelium toward a mesenchymal state, thus contributing to trachomatous scarring. To this end, we analyzed the expression of two TGFβ genes, known as main EMT inducers, using qRT-PCR (Figure 1A). Both TGFβ genes were significantly upregulated upon infection with *Ct*; TGFβ1 and TGFβ2 showed a 2- and 7-fold increase in mRNA, respectively. Besides TGFβ proteins, EMT can be induced through several other stimuli, which all act via different pathways (overview presented in Figure 1B). The presence and/or activation of some of the main downstream factors in the EMT-inducing network were determined by immunoblot analysis. Infection of HCjE cells with *Ct* resulted in a statistically significant increase in phosphorylation of JNK, p38, and ERK1/2, suggesting the activation of MAP kinases downstream of TGFβ receptors and RTKs. Activation of the canonical TGFβ pathway was suggested by a significant rise in phosphorylated Smad3. HCjE cells responded to *Ct* infection by augmented GSK3β-inhibitory phosphorylation and activation of PI3K/AKT, suggesting involvement of Wnt and integrin signaling. As EMT-inducing signaling pathways converge to the upregulation of specific EMT-related transcription factors, the increased mRNA expression of SNAIL1 and ZEB2, (Figure 1C), additionally supported the possibility of EMT promotion in HCjE cells after infection with *Ct*.

### E-cadherin is downregulated in response to *Ct* infection

**Figure 2:**
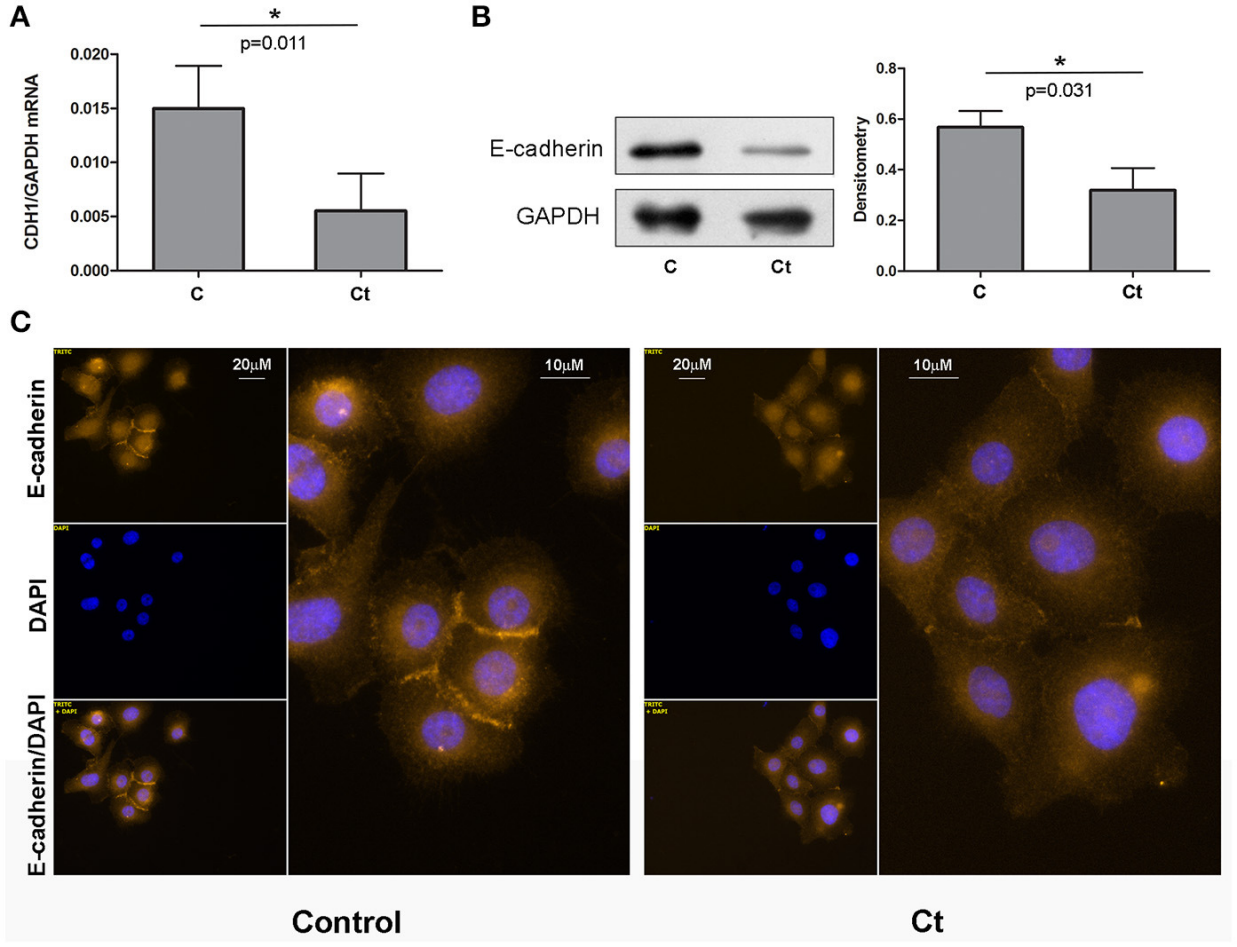
E-cadherin is Downregulated in Response to *Ct* Infection. **(A)** mRNA expression levels of *CDH1* in control (C) and HCjE cells after infection with 10^7^ IFU of *Ct* (Ct) as determined by qRT-PCR. mRNA levels are relative to that of *GAPDH* and are expressed as means ± SDs. **(B)** Protein levels of E-cadherin in control (C) and *Ct*-infected HCjE cells (Ct) as determined by immunoblot analysis using anti-E-cadherin antibody. Quantitative results of three immunoblots are presented, where E-cadherin protein levels are relative to the level of GAPDH, which was used as loading control. The results are expressed as means ± SDs. **(C)** Immunofluorescence analysis of control and *Ct*-infected HCjE cells with anti-E-cadherin antibody (light orange fluorescence). Nuclei are stained with DAPI (blue fluorescence).

As 72 h of infection of HCjE cells with *Ct* did not induce morphological change, but did trigger EMT-induced signaling, we next aimed to investigate whether *Ct* infection leads to EMT-related gene expression changes in HCjE cells, and to what extent. To this end, the mRNA and protein expression levels of E-cadherin were examined. Infection with *Ct* significantly reduced *CDH1* expression; the *CDH1* mRNA level was 2.7-fold higher in control than in *Ct*-infected HCjE cells (Figure 2A). Accordingly, E-cadherin protein was diminished 1.8-fold after *Ct* infection as revealed by immunoblotting (Figure 2B). Downregulation of E-cadherin after *Ct* infection was visualized and confirmed by immunocytochemistry; a marked loss of positive staining at cell–cell contacts after infection with *Ct* was observed (Figure 2C).

### *Ct* infection is accompanied by upregulation of mesenchymal markers α-SMA and fibronectin

**Figure 3:**
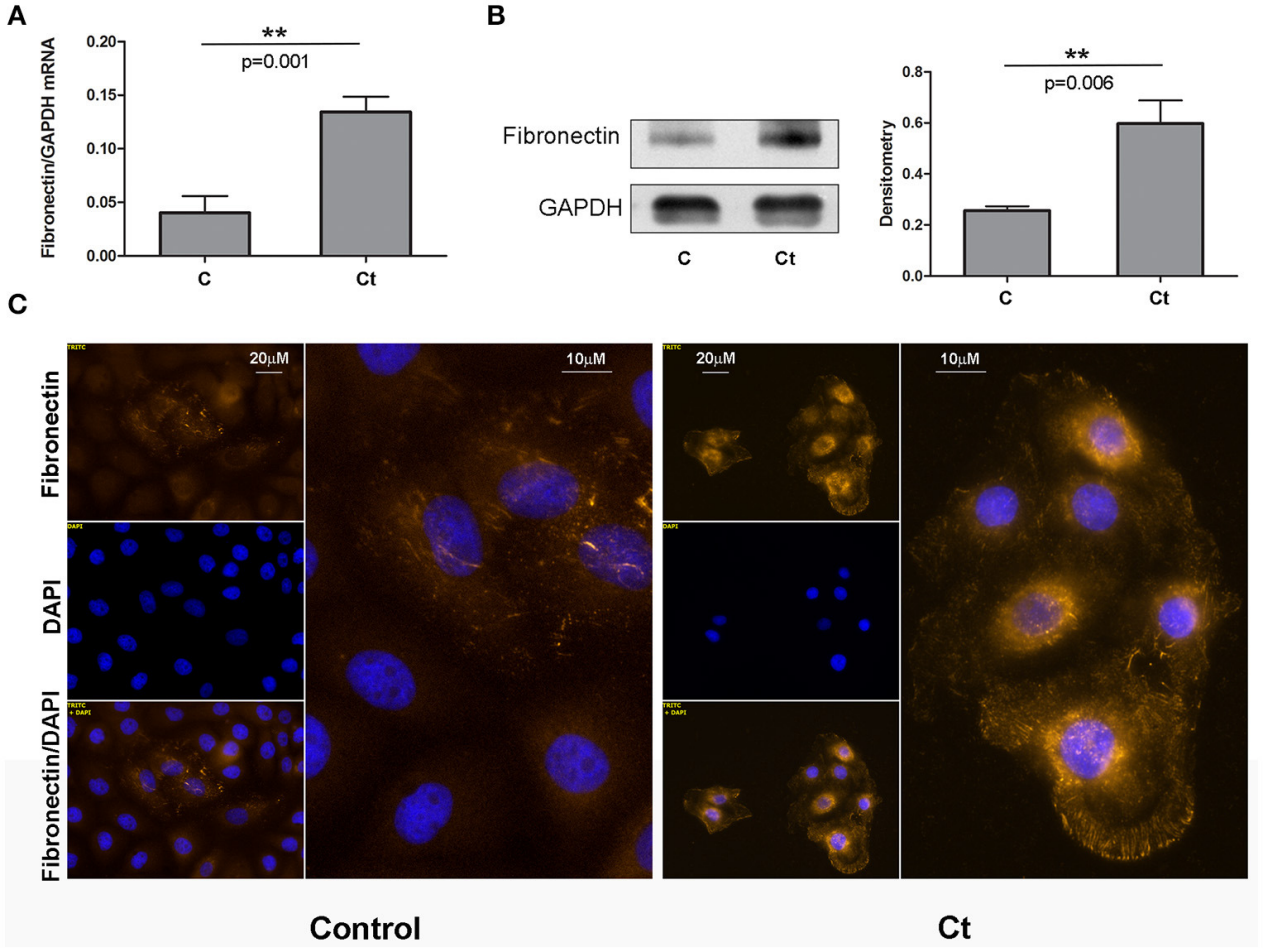
Mesenchymal Marker Fibronectin is Upregulated in Response to *Ct* Infection. **(A)** mRNA expression levels of *FN1* in control (C) and HCjE cells after infection with 10^7^ IFU of *Ct* (Ct) as determined by qRT-PCR. mRNA levels are relative to GAPDH and are expressed as means ± SDs. **(B)** Protein levels of fibronectin in control (C) and *Ct*-infected HCjE cells (Ct) as determined by immunoblot analysis using anti-fibronectin antibody. Quantitative results of three immunoblots are presented, where fibronectin protein levels are relative to the level of GAPDH, which was used as loading control. The results are expressed as means ± SDs. **(C)** Immunofluorescence analysis of control and *Ct*-infected HCjE cells with anti-fibronectin antibody (light orange fluorescence). Nuclei are stained with DAPI (blue fluorescence).

**Figure 4:**
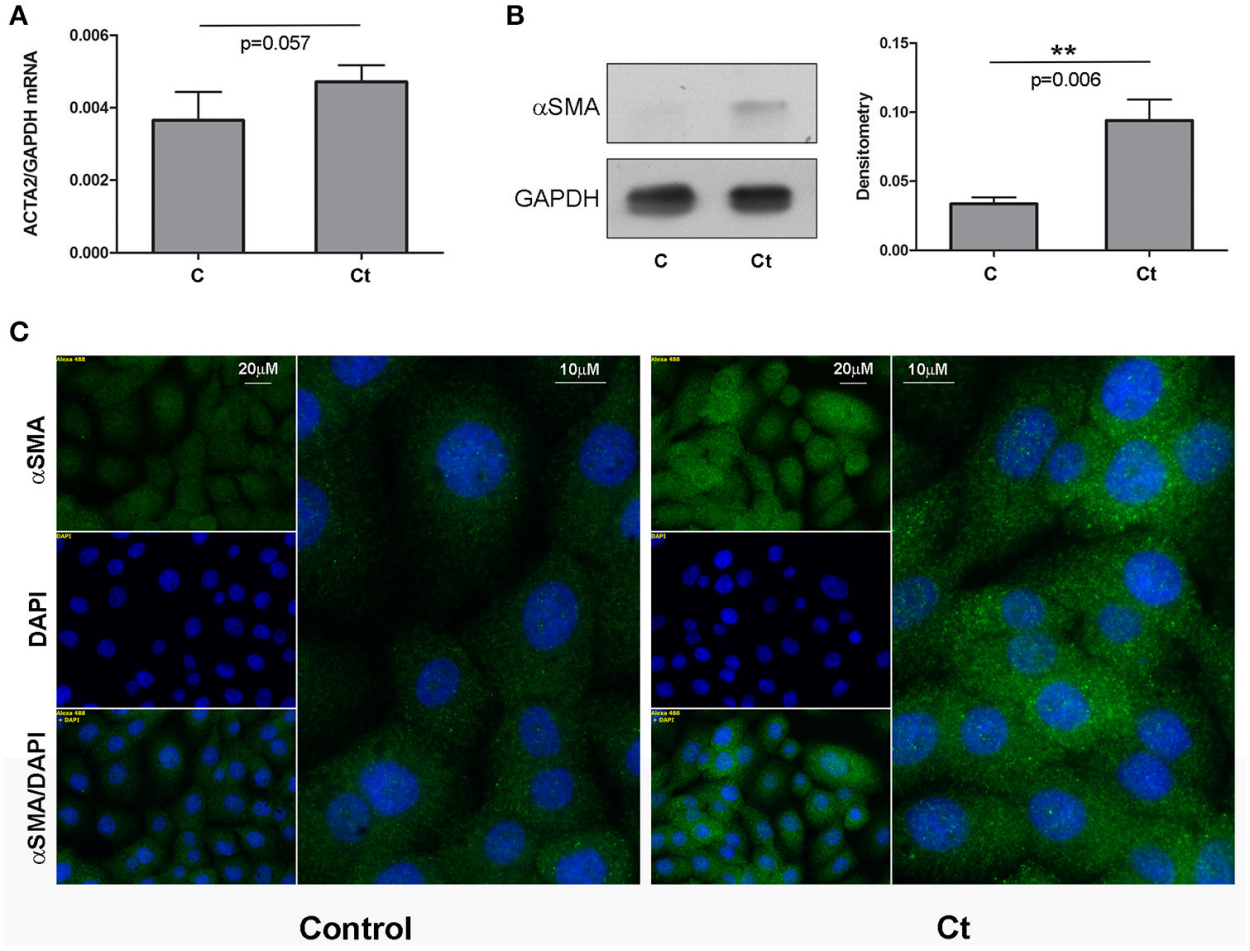
Mesenchymal Marker α-SMA is Upregulated in Response to *Ct* Infection. **(A)** mRNA expression levels of *ACTA2* in control (C) and HCjE cells after infection with 10^7^ IFU of *Ct* (Ct) as determined by qRT-PCR. mRNA levels are relative to that of *GAPDH* and are expressed as means ± SDs. **(B)** Protein levels of α-SMA in control (C) and *Ct*-infected HCjE cells (Ct) as determined by immunoblot analysis using anti-α-SMA antibody. Quantitative results of three immunoblots are presented, where α-SMA protein levels are relative to that of GAPDH, which was used as loading control. The results are expressed as means ± SDs. **(C)** Immunofluorescence analysis of control and *Ct*-infected HCjE cells with anti-α-SMA antibody (green fluorescence). Nuclei are stained with DAPI (blue fluorescence).

Next, we examined the effects of *Ct* infection on the expression levels of two mesenchymal markers, fibronectin and α-SMA, in HCjE cells (Figures 3, 4, respectively). *FN1* mRNA showed a statistically significant 3.3-fold increase (Figure 3A), while *ACTA2* mRNA was increased 1.3-fold in *Ct*-infected cells as compared to control cells (Figure 4A). Immunoblotting revealed 2.3- and 2.8-fold increases in fibronectin (Figure 3B) and α-SMA (Figure 4B), respectively, upon *Ct* infection. Immunocytochemistry with anti-fibronectin (Figure 3C) and anti-α-SMA antibodies (Figure 4C) confirmed the upregulation of both mesenchymal markers.

### DNA methylation status of *CDH1, FN1*, and *ACTA2* after *Ct* infection

**Figure 5:**
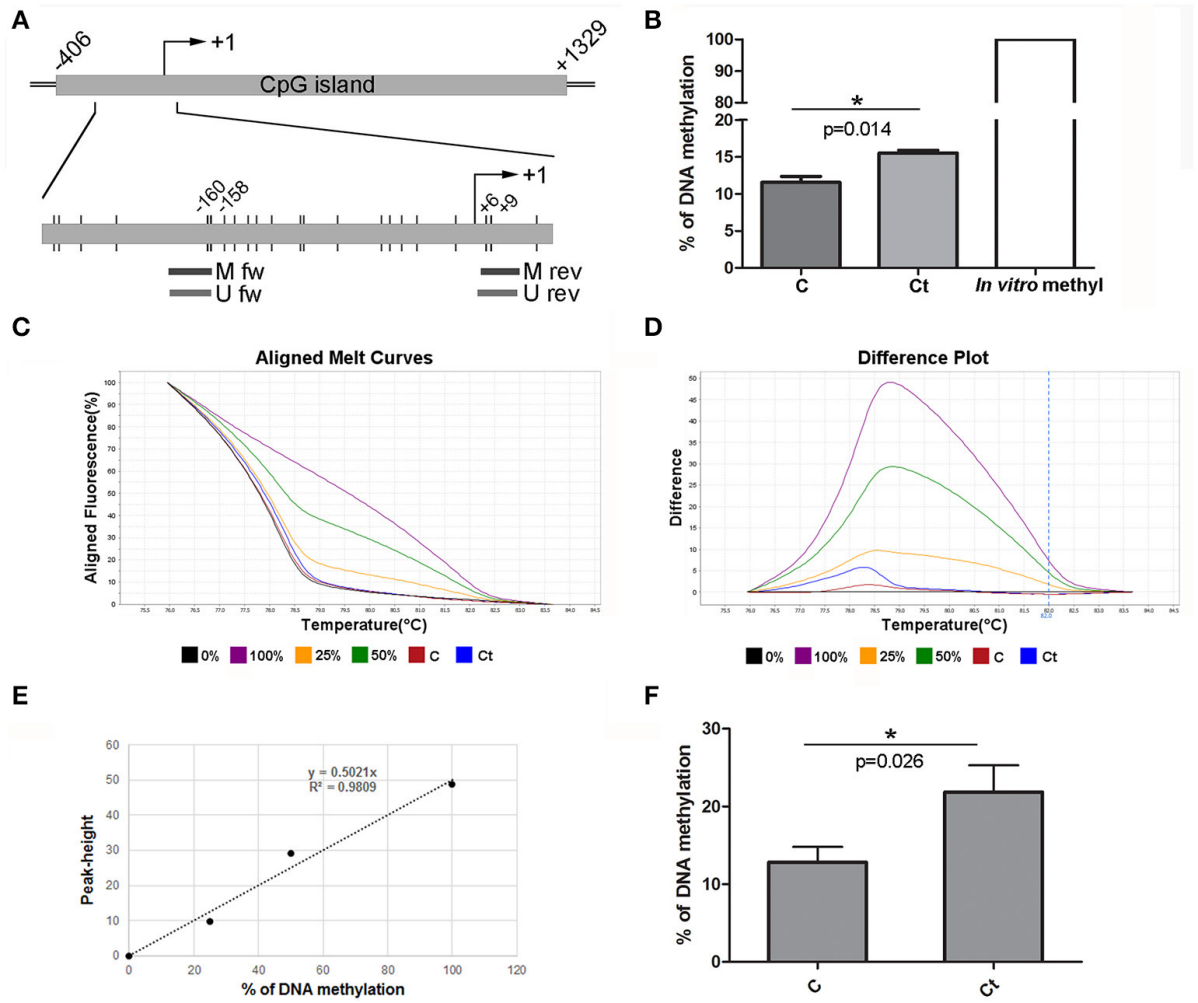
DNA Methylation Status of *CDH1* after Infection with *Ct*. **(A)** Schematic representation of part of the E-cadherin gene *CDH1* encompassing a CpG island. The position of the TSS is marked as “+1,” while the positions of primers used for DNA methylation analysis and of CpG dinucleotides (short vertical lines) are shown in the inset. M fw and M rev, methylated forward and reverse primers respectively; U fw and U rev, unmethylated forward and reverse primers, respectively. **(B)** Relative levels of methylated products obtained after MSP analysis of *CDH1* in control (C) and *Ct*-infected HCjE cells (Ct) compared to DNA methylated *in vitro*, assumed to be 100% methylated. The results are expressed as means ± SDs. **(C–F)** DNA methylation levels of the selected region obtained by HRM analysis. Representative aligned melt curves **(C)** and difference plots **(D)** showing positions of C and Ct curves with respect to 0, 25, 50, and 100% methylated standards. **(E)** Standard curve, plot of peak height versus percent of methylation (obtained as stated in the Materials and Methods section). **(F)** Bar graph depicting DNA methylation levels obtained from standard curves for three samples of each of control (C) and *Ct*-infected HCjE cells (Ct). The results are expressed as means ± SDs.

**Figure 6:**
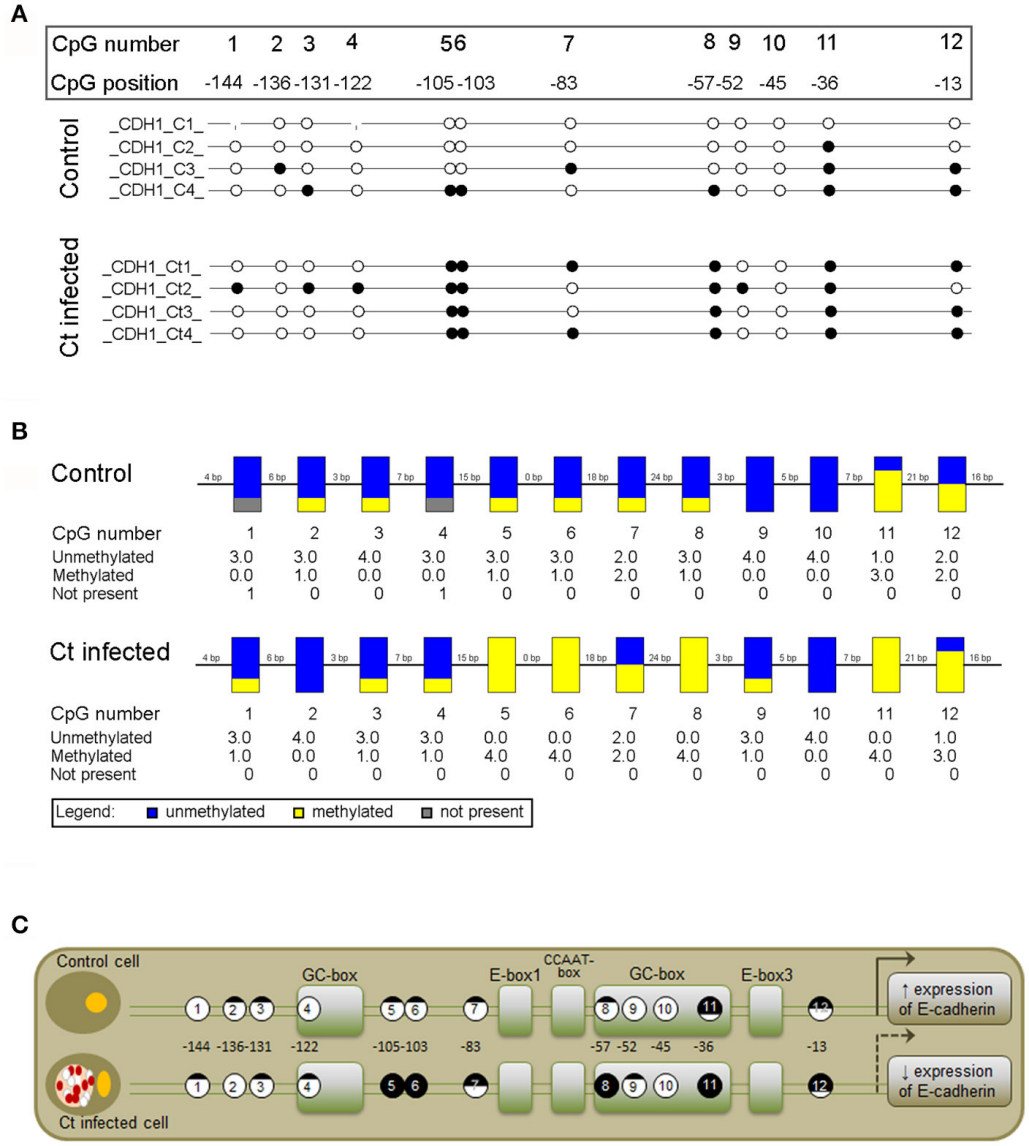
Detailed DNA Methylation Status of a Selected Promoter Region of *CDH1* after Infection with *Ct* Elucidated by Bisulfite Sequencing. **(A,B)** Schematic representation of the methylation status of a selected promoter region in control (C) and *Ct*-infected HCjE cells (Ct) obtained by Sanger sequencing. Bisulfite-converted DNA was subjected to PCR with methylated primers (shown in Figure 5A), and PCR products were sequenced using the corresponding forward primers. **(A)** Lollipop diagram representing methylation status of the promoter region of *CDH1* from four samples of each of C and Ct cells. The analyzed CpGs are numbered 1 to 12, and their positions with regard to the TSS are indicated. White circles, unmethylated CpG; black circles, methylated CpG. **(B)** Representation of combined DNA methylation data. Blue, unmethylated CpGs; yellow, methylated CpGs. **(C)** Positions and methylation status of CpGs in the context of regulatory elements present in the promoter region of *CDH1* (modified from van Roy and Berx, 2008).

To assess whether the observed changes in expression of the selected EMT markers are associated with altered DNA methylation of the corresponding genes, DNA methylation patterns of selected regions in the promoters and gene bodies of *CDH1, FN1*, and *ACTA2* were examined with MSP, MS-HRM, and bisulfite sequencing. The *CDH1* gene contains one large (1,735 bp) CpG island that covers the region from −406 to +1,329 with regard to the position of the TSS. The region selected for DNA methylation analysis encompasses the promoter sequence and TSS, and its genomic position, as well as those of the primers used in this study, is shown in Figure 5A. *Ct* infection initiated a 4% increase in DNA methylation, from 11.5% in control to 15.5% in *Ct*-infected cells (Figure 5B). The use of HRM allowed us to estimate the overall change in DNA methylation in the selected region (Figures 5C–F). HRM data revealed an increase from 12.8 to 21.8% after *Ct* infection, suggesting that the *CDH1* promoter region undergoes alterations in DNA methylation in HCjE cells as a result of *Ct* infection. To specify particular CpGs that are differentially methylated in control and *Ct*-infected cells, the selected region was subjected to bisulfite sequencing. Figure 6A shows the results of four samples. The sequenced region encompasses 12 CpGs (aside from CpGs positioned in primers), eight of which are in close proximity to the TSS. Of these, seven showed an increase in methylation level in *Ct*-infected cells. Moreover, methylation statuses at positions −57, −103, and −105 changed from 25% of methylation to fully methylated (100%) after *Ct* infection (Figure 6B).

**Figure 7:**
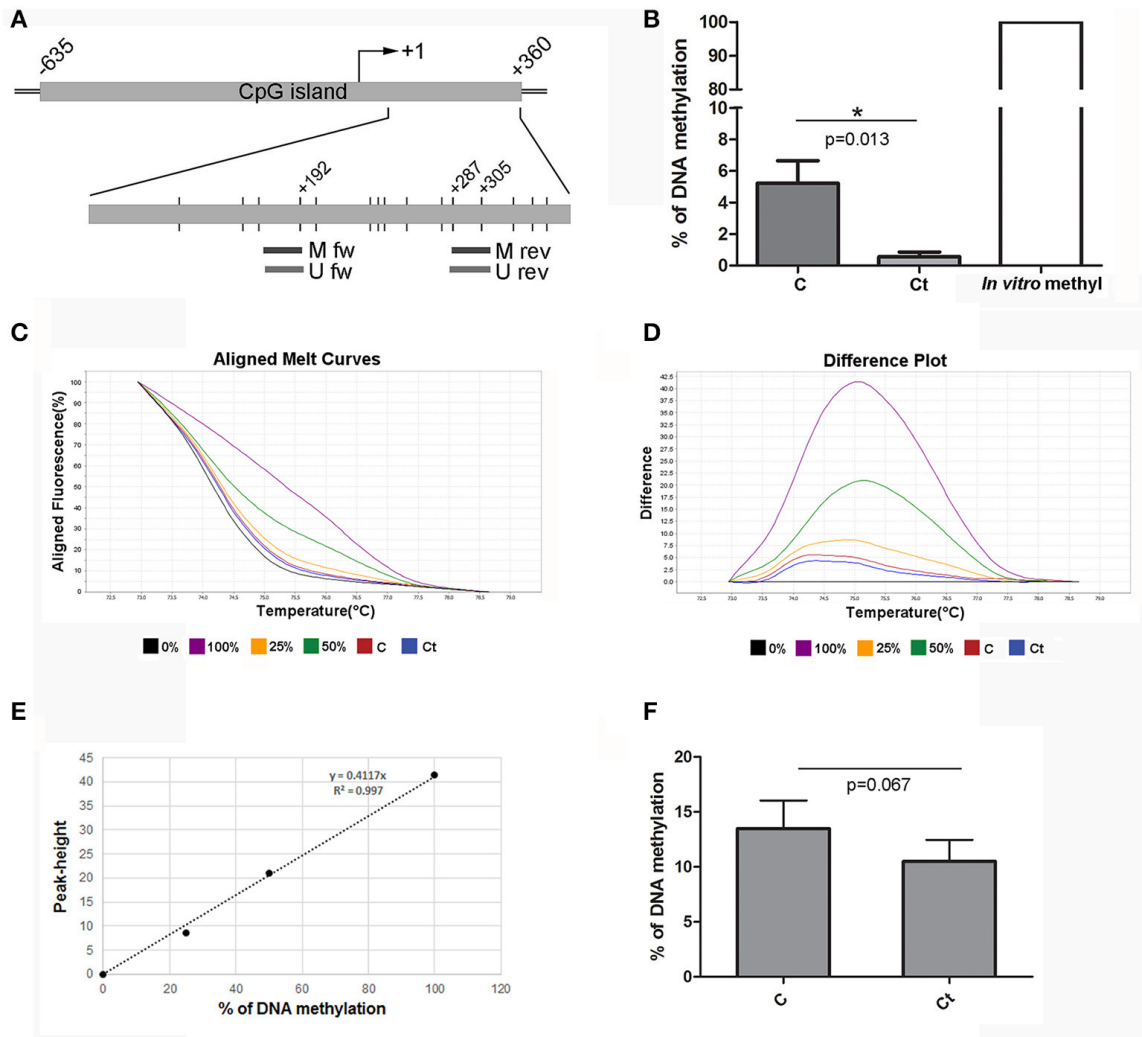
DNA Methylation Status of *FN1* after Infection with *Ct*. **(A)** Schematic representation of parts of the fibronectin gene *FN1* that encompass a CpG island. The position of the TSS is marked as “+1,” while the positions of primers used for DNA methylation analysis and of CpG dinucleotides (short vertical lines) are shown in the inset. M fw and M rev, methylated forward and reverse primers respectively; U fw and U rev, unmethylated forward and reverse primers, respectively. **(B)** Relative levels of methylated products obtained after MSP analysis of *FN1* in control (C) and *Ct*-infected HCjE cells (Ct) compared to DNA methylated *in vitro*, assumed to be 100% methylated. The results are expressed as means ± SDs. **(C–F)** DNA methylation levels of the selected region of *FN1* obtained by HRM analysis. Representative aligned melt curves **(C)** and difference plots **(D)** showing positions of C and Ct curves with respect to 0, 25, 50, and 100% methylated standards. **(E)** Standard curve, plot of peak height versus percent of methylation (obtained as stated in the Materials and Methods section). **(F)** Bar graph depicting DNA methylation levels obtained from standard curves for three samples of each of control (C) and *Ct*-infected HCjE cells (Ct). The results are expressed as means ± SDs.

The *FN1* gene contains one large CpG island (1,403 bp) located at –635 to +360 from the TSS. We selected a region within the gene body (first exon) for DNA methylation analysis. Positions of the selected region and primers are presented in Figure 7A. MSP analysis showed that approximately 5% of DNA from control HCjE cells was methylated in CpG positions located within the primer positions. However, after infection with *Ct*, these CpG positions were almost completely unmethylated (0.5% of methylation) (Figure 7B). HRM analysis revealed the same trend; DNA methylation decreased from 13.5% in control to 10.5% in *Ct*-infected HCjE cells (Figures 7C–F). However, we could not detect any difference in DNA methylation of *FN1* between control and *Ct*-infected HCjE cells by bisulfite sequencing, as the only methylated CpGs were positioned in primer sequences, while the other CpGs were all unmethylated in both samples (data not shown). Therefore, the MSP results reflect a precise quantification of changes in DNA methylation of the selected region. Based on the MSP and HRM data, it can be concluded that slight demethylation of 3–4% of the selected DNA region in *FN1* accompanies *Ct* infection.

**Figure 8:**
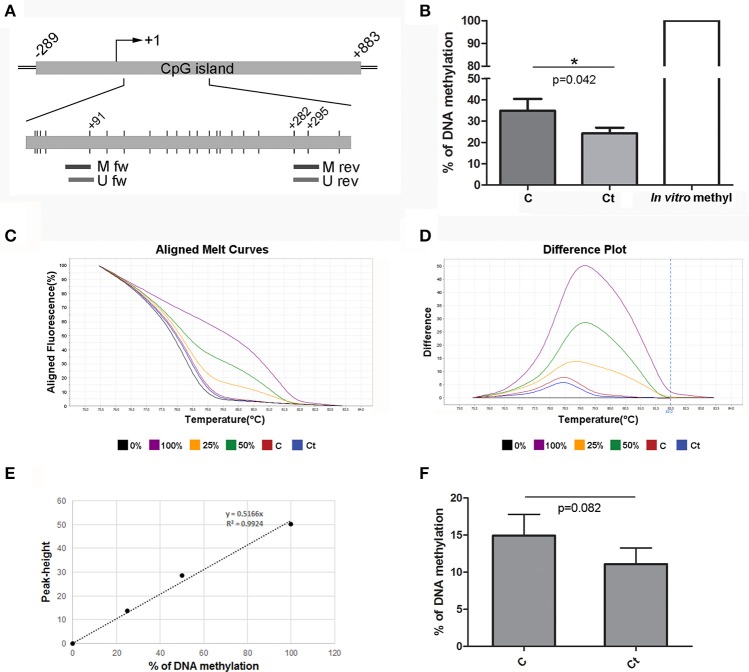
DNA Methylation Status of *ACTA2* after Infection with *Ct*. **(A)** Schematic representation of parts of *ACTA2*, which encompasses a CpG island. The position of the TSS is marked as “+1,” while the positions of primers used for DNA methylation analysis and of CpG dinucleotides (short vertical lines) are shown in the inset. M fw and M rev, methylated forward and reverse primers respectively; U fw and U rev, unmethylated forward and reverse primers, respectively. **(B)** Relative levels of methylated products obtained after MSP analysis of *ACTA2* in control (C) and *Ct*-infected HCjE cells (Ct) compared to DNA methylated *in vitro*, assumed to be 100% methylated. The results are expressed as means ± SDs. **(C–F)** DNA methylation levels of *ACTA2* selected region obtained by HRM analysis. Representative aligned melt curves **(C)** and difference plots **(D)** showing positions of C and Ct curves with respect to 0, 25, 50, and 100% methylated standards. **(E)** Standard curve, plot of peak height vs. percent of methylation (obtained as stated in the Materials and Methods section). **(F)** Bar graph depicting DNA methylation levels obtained from standard curves for three samples of each of control (C) and *Ct*-infected HCjE cells (Ct). The results are expressed as means ± SDs.

The *ACTA2* gene contains a CpG island located at –289 to +883 from the TSS. The region that was selected for DNA methylation analysis of *ACTA2* is located in the gene body (first exon), as shown in Figure 8A. MSP analysis revealed 10% diminished DNA methylation at CpG positions located within the primer positions in infected HCjE cells (Figure 8B). HRM analysis confirmed that infection of HCjE cells with *Ct* results in a decrease in *ACTA2* methylation, although to a lower extent than in MSP analysis (from 15% in control to 11% in *Ct*-infected cells) (Figures 8C–F). Bisulfite sequencing revealed that the entire assayed region (except CpGs in primer sequences) was completely unmethylated, without a difference in DNA methylation between control and *Ct*-infected cells (data not shown), indicating that MSP results give an adequate estimation of the *ACTA2* DNA methylation level in the selected region.

## Discussion

The importance of EMT in trachomatous scarring has been contemplated based on the finding that active trachoma is associated with increased expression of profibrotic cytokines, such as CTGF and IL-17A, which are known for their capability to induce EMT (Burton et al., 2011). TGFβ is an archetypal profibrotic molecule and the main EMT inducer; however, its role in trachomatous scarring remains unresolved. Some studies have reported constitutive expression of TGFβ proteins in the conjunctiva, without any difference between trachoma cases and controls (Burton et al., 2004; Derrick et al., 2016), while others have reported stimulation of TGFβ1 in the conjunctiva of trachoma patients (Bobo et al., 1996). We found that *Ct* infection strongly induces mRNA expression of TGFβ, particularly TGFβ2, in HCjE cells, in line with the finding that lung epithelial cells show enhanced secretion of TGFβ after infection with *Ct* (Williams et al., 1996). Viral infections of epithelial cells have also been shown to result in increased TGFβ production (Dosanjh, 2006; Park et al., 2014), suggesting that induction of TGFβ expression is a common response of epithelial cells to infection. The inconsistency in TGFβ expression data in trachoma might be explained by the presence or absence of infectious agents, as active trachoma is associated with the concomitant inflammation rather than *Ct* infection itself.

Besides the stimulation of TGFβ expression, infection of HCjE cells with *Ct* triggered the activation of a multitude of signaling pathways which are also, but not exclusively, downstream of major EMT inducers. Activation of the PI3K pathway with consecutive phosphorylation of AKT and its downstream target BAD is involved in the mechanism protecting *Ct*-infected host cells from apoptosis (Verbeke et al., 2006). MEK1/2 induction with subsequent ERK1/2 activation is implicated in promoting the acquisition of host phospholipids during the developmental cycle of *Ct* in the host cell (Su et al., 2004). Activation of the Wnt signaling pathway via GSK3β and subsequent sequestration of β-catenin away from the junctional complexes to *Ct* inclusion has been described in cervical epithelial cells infected with *Ct* (Prozialeck et al., 2002). In summary, none of these pathways are specific and exclusively related to EMT induction, but their joint action leads to upregulation of EMT-related transcription factors and might induce EMT in HCjE cells. The EMT induced after 72 h of *Ct* infection was only partial, as morphological change of HCjE cells was not observed.

The hallmark of the EMT process is a reduction in or loss of expression of E-cadherin, which is a guardian of the epithelial phenotype. We found decreases in mRNA and protein levels of E-cadherin. At the same time, *Ct* infection induced α-SMA and fibronectin expression, indicating that HCjE cells were moving toward a mesenchymal phenotype. The rise in fibronectin production is particularly significant, as it has been shown that fibronectin acts as a potent EMT inducer. Epithelial cells grown in the presence of fibronectin undergo spontaneous EMT via the activation of integrin signaling and integrin-dependent activation of endogenous latent TGFβ1 (Fontana et al., 2005; Kim et al., 2006). Thus, the observed enhancement of fibronectin production in *Ct*-infected HCjE cells might be an additional trigger of the EMT process in HCjE cells. The capability of *Ct* to induce changes in gene expression that are consistent with EMT induction in conjunctival epithelial cells is in line with the recent finding that *Ct* infection can induce EMT in reproductive epithelial cells *in vitro*, as indicated by downregulation of the epithelial markers E-cadherin and occludin and upregulation of the mesenchymal markers SNAIL1/2, fibronectin, MMP9, T-cadherin, and ZEB1 in these cells (Igietseme et al., 2015). Additionally, the authors found evidence of EMT induction by *Ct* infection *in vivo*, linking trachomatous scarring *in vivo* to EMT triggered by *Ct* infection.

Downregulation of E-cadherin expression is a highly complex process that is carried out through DNA methylation of its promoter and transcriptional repression (Strathdee, 2002). CpG hypermethylation is associated with the recruitment of methyl-CpG-binding proteins and consequent recruitment of histone deacetylase enzymes, leading to histone H3 deacetylation and E-cadherin transcriptional suppression (Koizume, 2002). We analyzed the DNA methylation status of the well-defined *CDH1* promoter subregion, the sequence located in close proximity to the TSS, as it directly correlates with E-cadherin expression (Reinhold et al., 2007). The increase in DNA methylation from 12.8% in control cells to 21.8% after *Ct* infection should be critically important for E-cadherin expression, as it has been shown that a sharp decline in *CDH1* expression occurs above 15% of promoter methylation (Reinhold et al., 2007). Bisulfite sequencing revealed that CpGs at positions −57, −103, and −105 are fully methylated after *Ct* infection, suggesting that those CpGs could be the first sites to be methylated in response to infection with *Ct* and particularly important for *CDH1* expression because of their position in the context of promoter-regulatory elements (Figure 6C). Direct transcriptional control of E-cadherin has emerged as an important regulatory mechanism in recent years. The *CDH1* promoter region selected for analysis contains positive regulatory elements as a CCAAT motive and two GC-rich boxes, recognized by AP2 and SP1 transcription factors involved in basal E-cadherin expression (Hennig et al., 1996; Faraldo et al., 1997). One of the fully methylated CpGs (position −57) is part of a GC-rich box, and its methylation could interfere with binding of transcription factors and thus influence *CDH1* transactivation. The *CDH1* promoter also contains negative regulatory E-box elements required for active suppression of E-cadherin expression (van Roy and Berx, 2008). E-cadherin suppression is mediated by EMT-related transcription factors such as ZEB1/2, TWIST, and SNAIL proteins (Thiery et al., 2009) through the recruitment of various histone methyltransferases, deacetylases, and demethylases, each responsible for a specific particular histone modification (Serrano-Gomez et al., 2016). In addition, it has been shown that SNAIL and ZEB1 cooperate with DNA methyltransferases (Lin et al., 2014; Fukagawa et al., 2015), allowing in-site DNA methylation besides histone modifications. This could be the immediate cause of the increased methylation of CpGs adjacent to E-boxes that we observed in *Ct*-infected cells. Together, our results implicate that DNA methylation might be an additional mechanism contributing to transcriptional repression of E-cadherin expression after *Ct* infection.

Although it is well accepted that DNA methylation of the promoter region strongly correlates with transcriptional repression, DNA methylation downstream of the TSS, in particular of the first exon, is most critical for transcriptional silencing, independently of the cell type (Brenet et al., 2011). Highly methylated DNA sequences in the rat α-SMA gene body correlated with its transcriptional suppression, while the level of promoter methylation did not vary between α-SMA-expressing and non-expressing cells (Hu et al., 2010). Therefore, we selected sequences within the first exon for analysis of DNA methylation of α-SMA and fibronectin. The results revealed a decrease in the α-SMA methylation level after *Ct* infection of 4 or 10%, with the only methylated CpGs located within the primer sequences. Thus, we cannot state that the decrease in gene methylation significantly contributed to the observed increase in mRNA and protein expression of α-SMA, at least with regard to the selected region and time after *Ct* infection. Based on our results, the augmentation of fibronectin expression on both the mRNA and the protein level after *Ct* infection did not result from the decrease in DNA methylation of the fibronectin gene. Even though we found a slight decrease in DNA methylation of 3 and 4% after *Ct* infection in DNA methylation status using MSP and HRM, respectively, bisulfite sequencing revealed that the selected region was completely unmethylated. These results are in agreement with a previous study investigating changes in fibronectin expression levels in relation to DNA methylation and histone acetylation (Kicic et al., 2010). By examining DNA methylation in two CpG islands upstream and downstream of TSS, the authors found no evidence of DNA methylation in circumstances of low fibronectin expression (Kicic et al., 2010).

Our results indicate that EMT could be an additional underlying mechanism of fibrotic processes in trachoma. Using an *in vitro* model of conjunctival *Ct* infection over 72 h, we could detect the earliest EMT-related events, such as activation of EMT-related signaling pathways, followed by up- and down-regulation of specific EMT markers. However, within the observed timeframe of 72 h, complete transition toward a fibrotic phenotype, including morphological changes, were not observed in the applied *in vitro* model. Thus, an *in vitro* experiment with long-term antibiotics treatment of HCjE cells, 72 h post *Ct* infection, will be conducted to prolong the lifespan of the cells and open a timeframe for analysis of induced phenotypic changes and their transgenerational transmission. Furthermore, additional *in vivo* studies are needed to unequivocally confirm the involvement of EMT in trachomatous scarring. However, this study presents a solid foundation for further functional analysis of DNA methylation during *Ct* infection, given that the results presented here are the first, to our knowledge, to link *Ct* infection with modulation of host DNA methylation status. A genome-wide DNA methylation study revealed that infection with another bacterium from the genus *Chlamydia, Chlamydia psittaci*, might be responsible for 180 differentially methylated genes in patients with ocular adnexal extranodal marginal zone B-cell lymphoma (EMZL) (Lee et al., 2014). Moreover, *Ct* produces NUE methyltransferase, which is able to methylate mammalian histones (Pennini et al., 2010), and alters the expression of crucial miRNAs that control EMT and fibrosis (Igietseme et al., 2015). Additionally, differential expression of several miRNAs has been described in trachoma patients (Derrick et al., 2013, 2016). Taken together, the data point to a straightforward link between *Ct* infection and a spectrum of epigenetic changes in the host cell.

If confirmed, our finding that *Ct* infection might induce EMT in host cells through DNA methylation changes could play a particularly important role in the development of new therapeutic approaches to trachoma treatment, based on the reversibility of both processes of EMT and DNA methylation. Targeting *Ct*-induced EMT by demethylating agents, such as the DNA methyltransferase inhibitor azacitidine, might have significant therapeutic potential. However, the main obstacle of epigenetic drugs is their complex network of functions and concomitant variety of side effects associated with systemic application. This could be avoided using gene editing by targeted DNA demethylation and activation of the E-cadherin gene that is essential for maintaining epithelial cell identity. This approach seems as a promising methodology for use in trachoma treatment, as it would potentially enable reversing or at least stopping scar progression in ocular *Ct* infection.

## Author contributions

Initiated the project: TB. Conceptualization: TB, AI, MV, ES, and NG. Conceived and designed the experiments: NG, MV, AI, ES, and TB. Performed the experiments: JR, NG, ES, AI, SD, NS, AU, EG, and MM. Analyzed the data: JR and NG. Contributed reagents/materials/analysis tools: TB and MV. Writing – original draft: JR, NG, MV, AI, ES, and TB. Writing – review and editing: JR, AI, NG, ES, SD, NS, AU, EG, MM, MV, and TB.

### Conflict of interest statement

The authors declare that the research was conducted in the absence of any commercial or financial relationships that could be construed as a potential conflict of interest.
