# Supplementary material for: Chlamydia trachomatis Infection Is Associated with E-Cadherin Promoter Methylation, Downregulation of E-Cadherin Expression, and Increased Expression of Fibronectin and α-SMA—Implications for Epithelial-Mesenchymal Transition
Source: Front Cell Infect Microbiol. 2017 Jun 14;7:253. doi: 10.3389/fcimb.2017.00253 (PMC5469886; doi:10.3389/fcimb.2017.00253)
Supplement: Supplementary file 1 [file Table1.pdf]

Table S1

Primer sequences used for qRT-PCR

| Gene name | Forward primer (5′-3′) | Reverse primer (5′-3′) | Length of PCR product |
|---|---|---|---|
| *TGFβ1* | TACTACGCCAAGGAGGTCAC | CACGTGCTGCTCCACTTTTA | 198 bp |
| *TGFβ2* | CAAGAGCAGAAGGCGAATGG | TAGCAGGAGATGTGGGGTCT | 275 bp |
| *CDH1* | CTTTGACGCCGAGAGCTACA | CACACCATCTGTGCCCACTT | 154 bp |
| *FN1* | GCTGGGCGAGGGAGAATAA | ACCACATAGGAAGTCCCAGC | 209 bp |
| *ACTA2* | CTACGTGGGTGACGAAGCA | TGCTCTTCAGGGGCAACAC | 147 bp |
| *SNAI1* | CAAGGATCTCCAGGCTCGAA | TGACATCTGAGTGGGTCTGG | 266 bp |
| *ZEB2* | AAGTACCAGCGGAAACAAGGA | AGTGCTCGATAAGGTGGTGC | 277 bp |
| *GAPDH* | CGGAGTCAACGGATTTGGTCGTAT | AGCCTTCTCCATGGTGGTGAAGAC | 307 bp |
