# Supplementary material for: Chlamydia trachomatis Infection Is Associated with E-Cadherin Promoter Methylation, Downregulation of E-Cadherin Expression, and Increased Expression of Fibronectin and α-SMA—Implications for Epithelial-Mesenchymal Transition
Source: Front Cell Infect Microbiol. 2017 Jun 14;7:253. doi: 10.3389/fcimb.2017.00253 (PMC5469886; doi:10.3389/fcimb.2017.00253)
Supplement: Supplementary file 2 [file Table2.PDF]

Table S2

Antibodies used for Immunoblot analysis and Immunocytochemistry

| | | Dilution | Cat.No./ Manufacturer |
|---|---|---|---|
| | **Immunoblot analysis** | | |
| Primary Ab | anti-E-cadherin | 1:4000 | H-108/Santa Cruz Biotechnology |
| | anti-Fibronectin | 1:1000 | H-300/Santa Cruz Biotechnology |
| | anti-Smooth Muscle Actin | 1:1000 | CGA7/Santa Cruz Biotechnology |
| | anti-SMAD 2/3 | 1:2000 | FL-425/Santa Cruz Biotechnology |
| | anti-p-SMAD3 (Ser208) | 1:1000 | sc-130218/Santa Cruz Biotechnology |
| | anti-P38α | 1:1000 | C-20/Santa Cruz Biotechnology |
| | anti-p-P38 (Tyr182-R) | 1:1000 | sc-7975-R/Santa Cruz Biotechnology |
| | anti-JNK1 | 1:750 | F-3/Santa Cruz Biotechnology |
| | anti-pJNK (Thr183/Tyr185-R) | 1:750 | sc-12882-R/Santa Cruz Biotechnology |
| | anti-GSK-3β | 1:2000 | H-76/Santa Cruz Biotechnology |
| | anti-pGSK-3β (Ser9-R) | 1:1500 | sc-11757-R/Santa Cruz Biotechnology |
| | anti-GAPDH antibody | 1:1500 | FL-335/Santa Cruz Biotechnology |
| | anti-PI3 Kinase p110γ | 1:1000 | #4252/Cell Signaling Technology |
| | anti-Akt | 1:2000 | #9272/Cell Signaling Technology |
| | anti-phospho-Akt (Ser473) | 1:1000 | 193H12; #4058/Cell Signaling Technology |
| | anti-p44/42 MAPK (ERK1/2) | 1:2000 | 137F5; #4695/Cell Signaling Technology |
| | anti-phospho-p44/42 MAPK (pERK1/2) (Thr202/Tyr204) | 1:1000 | #9101 /Cell Signaling Technology |
| Sec. Ab | bovine anti-rabbit IgG-HRP | 1:2000 | sc-2379/Santa Cruz Biotechnology |
| | anti-mouse IgG-HRP | 1:2000 | #7076/Cell Signaling Technology |
| | **Immunocytochemistry** | | |
| Primary Ab | anti-E-cadherin | 1:100 | H-108/Santa Cruz Biotechnology |
| | anti-Fibronectin | 1:150 | H-300/Santa Cruz Biotechnology |
| | anti-Smooth Muscle Actin | 1:100 | CGA7/Santa Cruz Biotechnology |
| Sec. Ab | goat anti-rabbit IgG, F(ab′)2-TRITC | 1:100 | sc-3841/Santa Cruz Biotechnology |
| | rabbit F(ab')2 anti-mouse IgG H&L-Alexa Fluor 488 | 1:100 | ab169345/Abcam |
