# Supplementary material for: Chlamydia trachomatis Infection Is Associated with E-Cadherin Promoter Methylation, Downregulation of E-Cadherin Expression, and Increased Expression of Fibronectin and α-SMA—Implications for Epithelial-Mesenchymal Transition
Source: Front Cell Infect Microbiol. 2017 Jun 14;7:253. doi: 10.3389/fcimb.2017.00253 (PMC5469886; doi:10.3389/fcimb.2017.00253)
Supplement: Supplementary file 3 [file Table3.pdf]

Table S3

Primer sequences used for DNA Methylation Analysis

**Gene name**		**Forward primer (5′-3′)**	**Reverse primer (5′-3′)**	**Length of PCR product**
CDH1	methylated primers (M)	GTAATTTTAGGTTAGAGGGTTATCGC	CACAAATACTTTACAATTCCGACG	212 bp
FN1		AAAACATTAAAAAAAATTTTTCCCG	TTGGACGGTTAGTAGTAGTAGTTCG	143 bp
ACTA2		TTTTTAGGTTCGGGTGTTTAGAAC	TATCAAAACTTATCCAAAAATTCCG	239 bp
CDH1	unmethylated primers (U)	ATTTTAGGTTAGAGGGTTATTGTGT	ACAAATACTTTACAATTCCAACACC	208 bp
FN1 U		AACATTAAAAAAAATTTTTCCCACA	TTGGATGGTTAGTAGTAGTAGTTTGG	141 bp
ACTA2		TTTTTAGGTTTGGGTGTTTAGAATG	TCAAAACTTATCCAAAAATTCCACT	237 bp
ACTA2	reference primers	TTTGAGAATTGTTAGAAAATGAGTAATATT	AACTCCTAAACAAACCCTAACAAAC	139 bp
